# Bioinformatics and Expression Analyses of miR-639, miR-641, miR-1915-3p and miR-3613-3p in Colorectal Cancer Pathogenesis

**DOI:** 10.7150/jca.86974

**Published:** 2023-07-31

**Authors:** Rusen Avsar, Turkan Gurer, Alper Aytekin

**Affiliations:** 1 Department of Biology, Faculty of Art and Science, Gaziantep University, 27310, Gaziantep, Turkey.; 2 Department of General Surgery, Faculty of Medicine, Gaziantep University, 27310, Gaziantep, Turkey.

**Keywords:** colorectal carcinoma, expression, microRNA, bioinformatics analysis, biomarker

## Abstract

**Objectives:** MicroRNAs (miRNAs) have important function in cancer development and progression. This study aims to determine the expression levels of miR-639, miR-641, miR-1915-3p, and miR-3613-3p in tissues of colorectal cancer (CRC) patients and the role of these miRNAs in the CRC pathogenesis.

**Methods:** Tumor and non-tumor tissues were collected from a total of 59 CRC patients. qRT-PCR was used to identify the expressions of miR-639, miR-641, miR-1915-3p and miR-3613-3p. Through bioinformatics analysis, the target genes of miRNAs were identified by using DIANA mirPath v.3. Signaling pathways were generated using KEGG pathway database. Biological pathway, cellular component analysis, and analysis of Protein-Protein Interactions (PPI) Networks were performed using FunRich and STRING database.

**Results:** Our findings revealed that miR-639, miR-641 and miR-3613-3p were significantly downregulated, and miR-1915-3p was significantly upregulated in tumor tissues compared to non-tumor tissues (*p*˂0.05). Furthermore, MAPK signaling pathway was the most enriched KEGG pathway regulated by miR-639, miR-641, miR-1915-3p and miR-3613-p. According to the FunRich, it was demonstrated that the targeted genes by miRNAs related to the cellular component and biological pathways such as beta-catenin-TCF7L2, axin-APC-beta-catenin-GSK3B complexes, Arf6 signaling, Class I PI3K signaling, etc. And, by the PPI analysis, it was established that the target genes were clustered on *CTNNB1* and *KRAS*.

**Conclusions:** These outcomes imply that miR-639, miR-641 and miR-3613-3p have tumor suppressor roles, while miR-1915-3p has an oncogenic role in the pathogenesis of CRC. According to the results of the current study, dysregulated miR-639, miR-641, miR-1915-3p, and miR-3613-3p might contribute to the development of CRC.

## Introduction

Colorectal cancer (CRC) is one of the most common malignant tumors and is the second leading cause of cancer-related deaths globally. According to the latest data, it is estimated that approximately 153,000 individuals will be diagnosed with CRC and 52,550 cases will die in 2023. Although many different diagnostic and treatment methods have been used so far, its incidence has been rising steadily, and it is not possible to prevent the progression of cancer in these patients 1, 2. In most cases, CRC is asymptomatic until it progresses to advanced stages. Thus, early diagnosis and improving screening programs would be critically important to reduce the incidence rates and mortality rates of CRC patients. Therefore, new biomarkers are needed to improve the diagnosis and treatment strategies of patients with CRC.

MicroRNAs (miRNAs) that are approximately 17-25 nucleotides in length are involved in the regulation of gene expression. Through binding to the 3' untranslated regions (3' UTR) of target mRNA molecules, miRNAs either degrade mRNA molecules or prevent the translation of these molecules, thus affecting multiple signaling pathways. miRNAs play a crucial role in various cancer-related biological processes such as development, differentiation, proliferation, cell death, and signal transduction 3. Due to their essential roles in carcinogenesis, miRNAs are classified as oncomirs or tumor suppressor miRNAs. While oncomirs are highly expressed gene regulators that inhibit the translation of tumor suppressor genes, tumor suppressor miRNAs are effective in the translation of mRNAs with oncogenic properties 4. Recent studies have revealed the relationship between miRNAs and many types of cancer including CRC 5. In many of the studies carried out so far, it has been reported that many miRNAs were identified as oncogenic miRNAs in CRC such as miR-17, miR-19a and miR-19b, while several other miRNAs, including miR-30a, miR-198, miR-485-3p and miR-4728-5p were found to play a tumor suppressor role in CRC 6-10. Taking all into consideration, identification of new miRNA biomarkers and revealing the roles of these miRNAs are critical steps in colorectal carcinogenesis.

Numerous studies have shown that miR-639 is localized on 19p13.12 is dysregulated in different cancer types such as liver 11, tongue squamous cell carcinoma 12, ovary 13, breast 14 and bladder 15. miR-641 is located on 19q13.2 chromosome region and is expressed at different levels in osteosarcoma 16, lung 17, cervical 18 and gastric 19 cancers. A great number of studies have demonstrated that miR-1915-3p, which is located on the 10p12.31 chromosome, is dysregulated in breast 20, lung 21 and thyroid 22 cancers. In addition, miR-3613-3p (on the 13q14.2 chromosome region) was found to be downregulated in breast 23, melanoma 24 and gastric 25 cancers. Motivated by previous studies, we aimed to determine the expression levels of miR-639, miR-641, miR-1915-3p, and miR-3613-3p in tissues with CRC. Furthermore, using bioinformatics analyses, it was purposed to examine the relationship between the genes targeted by dysregulated miRNAs and colorectal carcinoma, and to perform pathway enrichment, biological pathway, and cellular component analyses of these genes.

## Materials and methods

### Tissue samples

Between January 2017 and February 2020, we collected 59 pairs of fresh tumor and adjacent non-tumor tissue obtained from CRC patients who underwent surgical operation at Gaziantep University Hospital, Turkey. All patients in this study should meet the following criteria: (1) confirmation of CRC by pathological analysis, (2) no previous history of CRC or other malignant tumors, (3) no radiotherapy or chemotherapy, and (4) no other anti-tumor therapy before surgery. Before collection of the tissues, a written informed consent form was obtained from the participants of this study. The study was conducted in accordance with the Declaration of Helsinki, and the protocol was approved by the local ethics committee of Gaziantep University, Turkey (Ethical approved numbers: 2019/420 and 2019/421). All tissues were stored in RNAlater (Thermo Fisher Scientific, USA) at -80 ˚C.

### RNA extraction and cDNA synthesis

Following the instructions provided by the company, total RNA from fresh tissue specimens was extracted using mirVana ™ miRNA Isolation Kit (Invitrogen, USA). NanoDrop spectrometer was used to assess the quality and the quantity of RNA. The isolated RNA was stored at -80 °C. Thereafter, RNA was reverse transcribed into cDNA using miScript II RT Kit (Qiagen, USA) according to the manufacturer's instructions. The obtained cDNA samples were stored at -20 °C.

### Quantitative Real-time PCR (qRT-PCR)

Using 7500 Fast Real-Time PCR system (Applied Biosystems) with miScript SYBR Green PCR Kit (Qiagen, USA), we performed qRT-PCR analysis. While *U6* was used as an endogenous control, we analyzed the expression levels of miR-639, miR-641, miR-1915-3p and miR-3613-3p. The reactions were completed in triplicate. The sequences of primers are: miR-639: Forward 5'-ATCGCTGCGGTTGCGAGCGCTGT-3', Revers 5'-CGAGGAAGAAGACGGAAGAAT-3'; miR-641: Forward 5'-AAAGACATAGGATAGAGTCACCTC-3', Revers 5'-CGAGGAAGAAGACGGAAGAAT-3'; miR-1915-3p: Forward 5'-CCCCAGGGCGACGCGGCGGG-3', Revers 5'-CGAGGAAGAAGACGGAAGAAT-3', miR-3613-3p: 5'-ACAAAAAAAAAAGCCCAACCCTTC-3', Revers 5'-CGAGGAAGAAGACGGAAGAAT-3'; *U6*: Forward 5'-GCTTCGGCAGCACATATACTAAAAT-3'; Revers 5'-CGCTTCACGAATTTGCGTGTCAT-3'.

The 2 ^-ΔΔCt^ method was used for real-time PCR data analysis 26. For this method, we firstly determined fluorescence threshold cycle (Ct) of each sample. Then, ΔΔCt (ΔΔCt= ΔCt_tumor_ - ΔCt_control_) values ​​were calculated using ΔCt (ΔCt = Ct_target_ - Ct*_U6_*) values ​​obtained from tumor and healthy tissues. Finally, using 2^-ΔΔCt^ values, fold change values ​​were determined. The high expression level was defined as if those with fold change value above 1 while low expression as if those with fold change value below 1 10.

### Pathway and target gene analysis of miRNAs

DIANA-mirPath that can use predicted miRNA targets is a miRNA pathway analysis online tool. The target genes of miRNAs were identified by using DIANA mirPath v.3 with microT-CDS (v5.0) algorithm. A KEGG (Kyoto Encyclopedia of Genes and Genomes) analysis was accomplished on the genes targeted by miRNAs by using DIANA-mirPath v3.0 27 and DIANA-TarBase v7.0 28 databases. Signaling pathways and target genes of miRNAs involved in the formation of CRC carcinogenesis were generated using KEGG pathway database 29.

### Functional enrichment analysis

Functional enrichment analysis (FunRich) is a software package and this software is used for functional enrichment and interaction network analysis of genes and proteins 30. FunRich tool was used for biological pathway and cellular component analyses of genes targeted by miRNAs involved in CRC carcinogenesis, obtained using the DIANA database.

### Construction and analysis of protein-protein interactions (PPI) network

The Search Tool for the Retrieval of Interacting Genes (STRING) database is an online tool that generates direct and indirect connections between protein-protein interaction networks. The interactions between genes targeted by miRNAs were demonstrated with PPI network by using STRING database 31. A minimum required interaction score 0.4 was set as the cut-off criterion.

### Statistical analysis

All statistical analyses were performed using SPSS (version 22.0; IBM Corp.). The data was presented as the mean ± standard deviation. A paired t-test was used to test the difference in miRNA expression in tumor and adjacent non-tumor tissues by comparing ΔCt_tumor_ and ΔCt_control_ values. Furthermore, the association between miRNA expression levels and clinicopathological parameters was tested by Pearson chi-square (χ2) and Fishers' Exact tests. *p* <0.05 was considered a statistically significant level.

## Results

### Characteristics of patients with CRC

In this study, we used the tumors and adjacent healthy tissues from a total of 59 patients with CRC. While 59.3% of these patients were men, 40.7% of the patients were women. 66.1% of the patients were 55 years old and over (the median age: 55.06±11.89). 62.7% of the samples in the study were collected from the colon tissue and the rest from the rectum tissue. Based on patient medical history, 28.8% of the patients used to smoke, while 8.5% consumed alcohol. Lymph node metastasis was observed in 40.7% of all cases, and distant metastasis was detected in 16.9% of the patients. According to the American Joint Committee on Cancer (AJCC) TNM system, 50.8% of patients were detected in Stage I and II, and 49.2% in Stage III and IV. In terms of histological types, 72.9% of the tissues were adenocarcinoma and 27.1% were mucinous adenocarcinoma. The demographic, clinical and pathological data of the patients with CRC are summarized in Table 1.

### Significant differences of expression levels of miR-639, miR-641, miR-1915-3p and miR-3613-3p between tumor and adjacent non-tumor tissues

**Figure 1:**
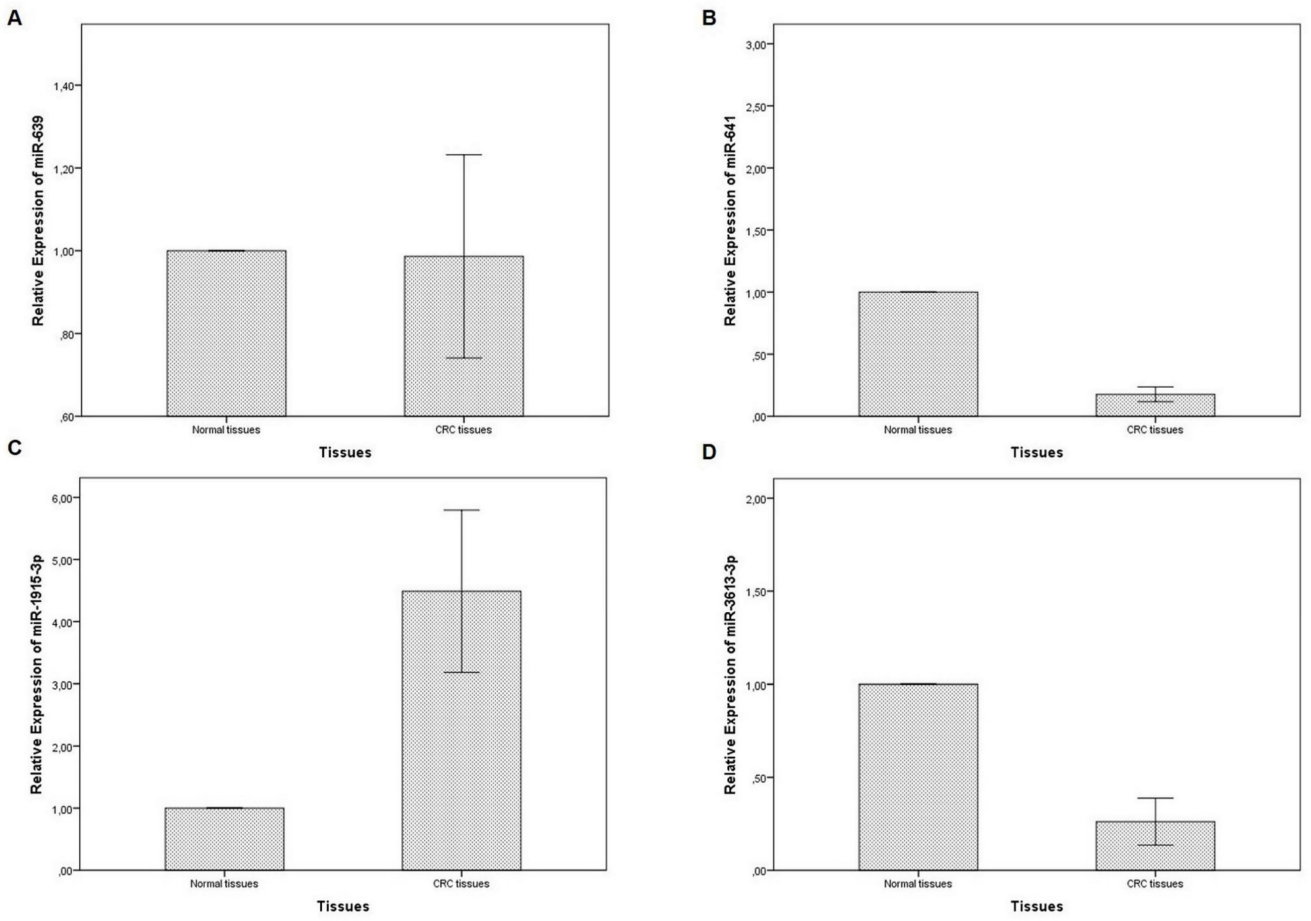
Expression levels of miR-639 (A), miR-641 (B), miR-1915-3p (C) and miR-3613-3p (D) in the tumor and adjacent non-tumor tissues of patients with CRC

In the present study, the expression levels of miR-639, miR-641, miR-1915-3p and miR-3613-3p were analyzed in the tumor and adjacent healthy tissues of a total of 59 patients with CRC. When compared to healthy tissues, miR-639 (*p*=0.000), miR-641 (*p*=0.000) and miR-3613-3p (*p*=0.000) (fold change values; 0.20±0.26, 0.17±0.22, and 0.26±0.48, respectively) were significantly downregulated in tumor tissues, whereas miR-1915-3p was significantly upregulated (*p*=0.032) (fold change value; 4.48±5.01) (Figure 1).

### The relationship between expressions of miR-639, miR-1915-3p and miR-3613-3p and clinicopathological features of patients with CRC

Our results showed that the decreased expression of miR-639 was significantly higher compared to the increased miR-639 expression in both colon and rectum tissues of patients (*p*=0.049). In patients with advanced-stage tumors (Stage III and IV), miR-639 was found to be significantly downregulated (*p*=0.001). However, we did not observe any significant association between miR-639 expression and other variables (*p*>0.05). We further did not find a significant relationship between the expressions of both miR-1915-3p and miR-3613-3p and the clinicopathological characteristics of the patients (*p*>0.05) (Table 2). miR-641 was excluded from these assessments since it was downregulated in all patients.

### Bioinformatics analyses

**Figure 2:**
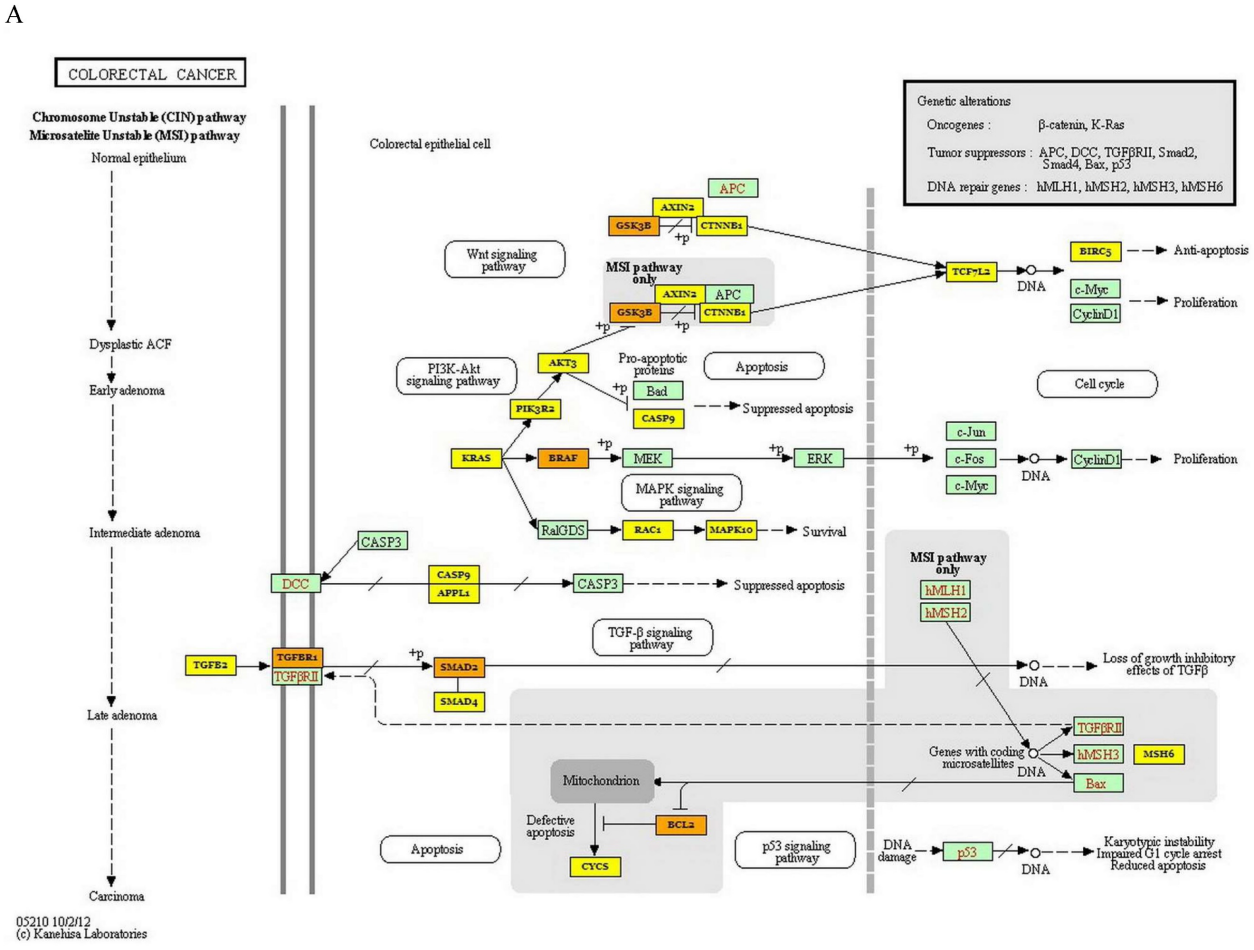

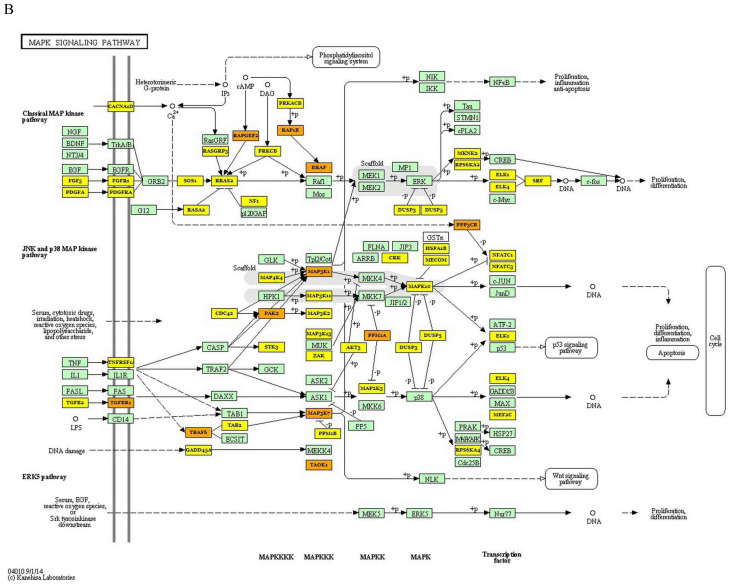
Kyoto Encyclopedia of Genes and Genomes pathway of target genes in CRC. A- Signaling pathways and the target genes associated with colorectal carcinogenesis. B- MAPK signaling pathway (the target genes given in the yellow and orange boxes (the genes targeted by 1 and more than 1 miRNAs, respectively) indicate the target genes in the colorectal carcinogenesis and MAPK signaling pathway)

KEGG analysis of 3 miRNAs was performed with DIANA miRpath online tool in this study. The target genes of each miRNA in CRC were determined. As a result of this analysis, it was determined that 24, 7 and 3 genes were targeted by miR-3613-3p, miR-641 and miR-1915-3p, respectively (*p* values: 4.483447∙10^-79^, 2.315548∙10^-19^ and 8.078544∙10^-8^, respectively). A total of 26 genes were found to be targeted by these 3 miRNAs in CRC (*p*=0.00808911396543) (Figure 2A). The target gene of miR-639, which is effective in colorectal carcinogenesis, was not found in the DIANA miRpath software. Furthermore, KEGG pathway analysis revealed that MAPK, Wnt, PI3K-Akt, p53, and TGF- signaling pathways were significantly enriched in the pathogenesis of CRC. Additionally, the most enriched KEGG pathway regulated by miR-639, miR-641, miR-1915-3p and miR-3613-p was the MAPK signaling pathway (*p*-value threshold=0.0022616148417), and *TGFB1, BRAF, MAP3K1, MAP3K7, PAK2, TAOK1, PPP3CB, RAPGEF2, TRAF6, CACNA2D1* and *RAP1B* were among the target genes of the pathway (Figure 2B).

**Figure 3:**
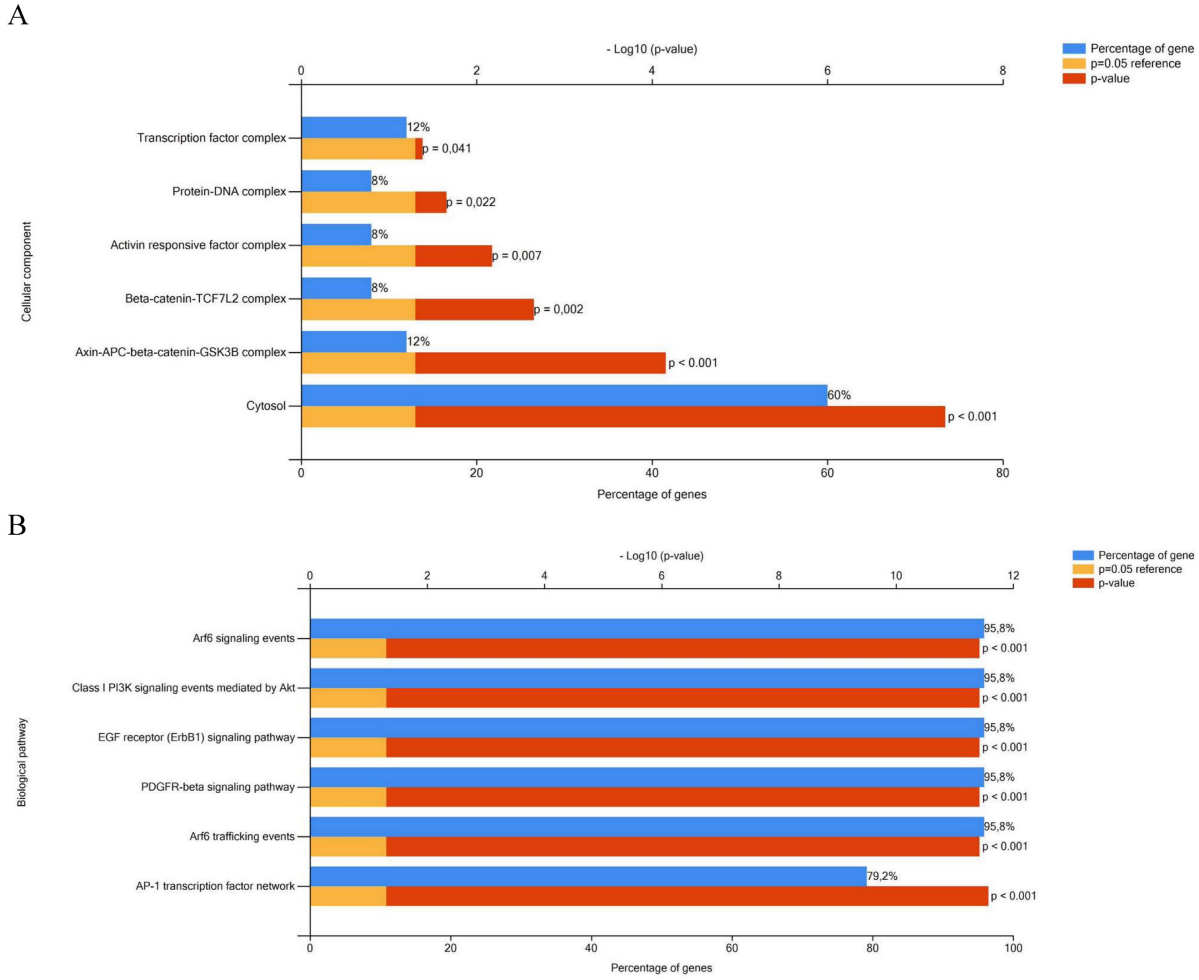
Functional enrichment analysis of the targeted genes by miRNAs in CRC. A- Cellular component analysis. B- Biological pathway analysis

Biological pathway and cellular component analyses of a total of 26 genes targeted by miR-641, miR-1915-3p and miR-3613-3p dysregulated in colorectal carcinogenesis were performed using FunRich enrichment assay. According to the FunRich enrichment analyses, it was seen that these genes related to transcription factor (12%, *p*=0.041), protein-DNA (8%, *p*=0.022), activin responsive factor (8%, *p*=0.007), beta-catenin-TCF7L2 (8%, *p*=0.022), axin-APC-beta-catenin-GSK3B (12%, *p*˂0.001) complexes and cytosol (60%, *p*˂0.001) among the cellular components (Figure 3A). As can be seen in the Figure 3B, these 26 targeted genes were involved in biological pathways such as Arf6 signaling (95.8%, *p*˂0.001), Class I PI3K signaling (95.8%, *p*˂0.001), EGF receptor (ErbB1) signaling (95.8%, *p*˂0.001), PDGFR-beta signaling (95.8%, *p*˂0.001), Arf6 trafficking (95.8%, *p*˂0.001) and AP-1 transcription factor network (79.2%, *p*˂0.001).

**Figure 4:**
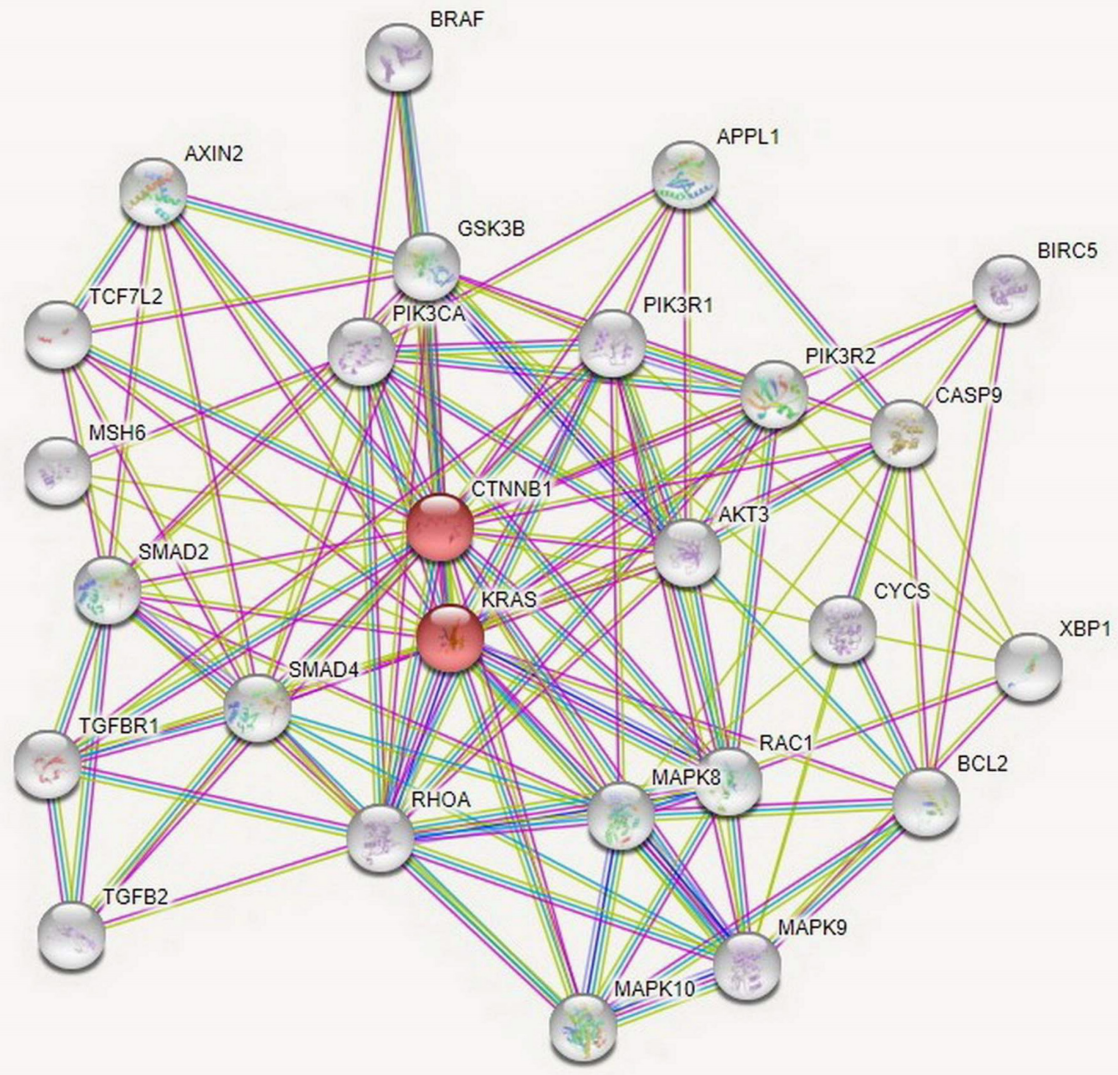
Protein-Protein Interactions networks of the targeted genes by miRNAs in CRC

The PPI network was comprised of 26 nodes and 136 edges, which were mapped by STRING software (Figure 4). It was observed that the average node degree was 10.5, expected number of edges was 34 and PPI enrichment *p*-value was < 1.0∙10^-16^. As a result of the PPI analysis, it was established that these 26 genes were clustered on *CTNNB1* and *KRAS*. This interaction between genes is thought to be effective in the regulation of cell proliferation in CRC.

## Discussion

CRC is the second leading reason of cancer-related deaths worldwide. Therefore, early diagnosis of the tumor is crucial for the survival period of patients with CRC. In this perspective, the identification of specific and strong molecular biomarkers for the early detection of CRC is highly essential 32. In recent years, there have been many studies showing that various miRNAs have an oncogene or tumor suppressor role in almost all human malignancies, including CRC. They also have an effect on tumor formation and progression and, therefore, are used as prognostic biomarkers 18-33. These studies highlight the importance of the dysregulation of miRNAs in the tumor formation process. Therefore, herein, we investigated the expression levels of miR-639, miR-641, miR-1915-3p, and miR-3613-3p in tissues with CRC. Also, in this study, we examined the biological pathways of the target genes of these miRNAs, their effects on colorectal carcinoma, and the interaction network between the proteins encoded by these genes using bioinformatics analysis tools.

In the current study, we found that miR-639 decreased significantly in CRC tissue samples compared to non-tumor tissue samples. In fact, similar results were reported for hepatocellular carcinoma, and miR-639 was shown to act as a tumor suppressor 34. In a previous study, Xiao et al. (2020) showed that the expression of miR-639 was decreased in liver cancer tissues and this decrease was due to the hypermethylation of the promoter region of miR-639 by DNMT3A. They further reported that miR-639 binds to the 3'-UTR regions of *MYST2* and *ZEB1* and suppresses the expression of these genes 11. Wu et al. (2021) demonstrated that expression of miR-639 that targeted *FOXC1* was downregulated in ovarian cancer 13. Moreover, our results reveal a significant relationship between the advanced stages of CRC (stage III and IV) and low miR-639 expression, indicating that miR-639 can be an important biomarker which can be used in the diagnosis and prognosis of CRC. Contrary to our study, miR-639 was previously found to have an oncogenic role in thyroid, bladder and breast cancers 14, 15, 35.

The present study indicated that the expression of miR-641 was decreased in all tissues with CRC significantly when compared to healthy tissues. Similarly, a previous study reported that expression of miR-641 was downregulated in lung cancer tissues and cell lines and also targets *MDM2*, which was defined as an oncogene 36. In another study, miR-641 was also shown to have low expression in cervical cancer and bind directly to the 3'-UTR region of *ZEB1*, a member of the deltaEF1 family of two-handed zinc-finger factors 18. Recently, Meng et al. (2020) showed that down-regulation of miR-641 increased cell proliferation in lung adenocarcinoma 17. Wang et al. (2019) reported that in gastric carcinoma tissues and cells, expression of miR-641 was decreased and miR-641 expression was negatively correlated with lncRNA OPI5-AS1 19. Likewise, miR-641 was also downregulated in osteosarcoma tissues and cells 16. These results also support the findings of the present study in that miR-641 plays a tumor suppressor role in CRC and may be a powerful marker that can be used in the diagnosis of CRC.

Our RT-qPCR results showed that miR-1915-3p was upregulated in tissues with CRC compared to healthy tissues. Similarly, it was shown in a previous study that high expression of miR-1915-3p was associated with infiltrative follicular variants of papillary thyroid cancer and might be a diagnostic factor in thyroid cancer 22. In another published study, it was indicated that miR‑1915‑3p that targeted *DRG2/PBX2*, played a role as a silencer of apoptosis in lung cancer, and this miRNA might be a therapeutic target in lung carcinoma 21. It is also known that miR-1915-3p plays a tumor suppressive role in non-small-cell lung cancer 37. Guo et al. (2018) showed that miR-1915-3p was upregulated in serum samples of breast cancer patients compared with healthy individuals and that miR-1915-3p might be a diagnostic marker for breast cancer by repression of DUSP3 20. These results are compatible with the finding of recent studies that miR-1915-3p, which also plays an oncogenic role, is downregulated in CRC.

miR-3613-3p was among miRNAs that we investigated, and the results herein showed that miR-3613-3p was downregulated in tissues with CRC. In parallel with our outcomes, Yan et al. (2018) reported that the expression of miR-3613-3p was decreased in SW480 and SW620, which are human colon cancer cell lines with different metastatic features 38. However, miR-3613-3p, defined as a tumor suppressor in gastric cancer, was shown to be downregulated in melanoma cell lines and targeted to CD7 24, 25. Chen et al. (2021) indicated that miR-3613-3p plays a tumor suppressor role in the development of breast cancer and miR-3613-3p might be a novel biomarker for breast carcinogenesis 23. In another study conducted in triple negative breast cancer, it was shown that miR-3613-3p plays a role in suppressing cancer formation 39. These data also corroborate the conclusion that miR-3613-3p plays a tumor suppressor role and may be a potential biomarker for CRC.

KEGG that contains information of gene regulatory pathways, is an important data source for the modelling of biological systems 29. In the enriched KEGG pathway analysis, we determined that the signaling pathway in which the target genes of the 3 miRNAs affect together was MAPK. The MAPK is a significant signaling pathway that regulates various biological processes associated with tumor proliferation, development, differentiation, migration and apoptosis, and this signaling pathway plays an important role in CRC carcinogenesis 40. In the present study, it was determined that BRAF, TAOK1, TRAF6 and PAK2, which were involved in the MAPK signaling pathway, were among the genes targeted by downregulated miR-641 and miR-6313-3p (Figure 2B). Previous studies showed that BRAF was effective in the formation of CRC and played an oncogenic role in this carcinogenesis 41, 42. Capra et al. (2006) demonstrated that increased expression of TAOK1 was associated with CRC 43. In the other studies, TRAF6 and PAK2 were upregulated in colon cancer 44, 45.

Furthermore, we concluded that Arf6 signaling, Class I PI3K signaling, EGF receptor (ErbB1) signaling, PDGFR-beta signaling, Arf6 trafficking and AP-1 transcription factor pathways were associated with dysregulated miRNAs. These pathways have an important role in oncogenesis and cancer progression, such as tumor growth, differentiation, invasion, migration, metastasis, apoptosis and poor survival 46-50.

In this study, as a result of PPI analysis, we determined that 26 genes targeted by miRNAs were clustered on *CTNNB1* and *KRAS*. CTNNB1, a transcriptional factor, was upregulated in colorectal cancer tissues 51. *KRAS*, known as the oncogene, is regulated by tyrosine kinase receptors and the KRAS protein is an essential part of the MAPK signaling pathway. Previous studies demonstrated that *KRAS* mutations led to uncontrollable cell growth, cell differentiation, metastasis and angiogenesis in CRC 52. As we discussed above, although some of the previous studies investigating the relationships of miR-639, miR-641, miR-1915-3p, and miR-3613-3p with different types of cancer have resulted in similar outcomes to our study, there have been also other studies that obtain contrary outcomes. These contradictory results may be mainly related to the heterogeneity of the cancer. However, it is important to highlight that miRNAs target many different mRNA molecules in the genome and some of these target molecules act as oncogenes while some as tumor suppressors. Therefore, it is not a surprising outcome that the same miRNAs play different roles in different cancer types depending on the mRNA molecules they target.

The present study has several limitations. First, we did not perform an experimental study to identify genes targeted by miRNAs in this study. Therefore, it may focus on identification of the target genes of miR-639, miR-641, miR-1915-3p and miR-3613-3p that are effective in CRC in future studies. Second, the sample size of this study may be limited to clearly demonstrating the relationship between clinicopathological characteristics of patients and expression levels of miRNAs. Third, the effects of miRNAs on CRC cell proliferation, invasion, metastasis, and apoptosis were not investigated in vitro. Although the current research has several limitations, the results of this study will guide future studies to reveal the genetic mechanisms that are effective in determining the relationship between colorectal carcinogenesis and miRNAs.

## Conclusions

As a result, our findings suggest that miR-639, miR-641, and miR-3613-3p play tumor suppressor roles in CRC, whereas miR-1915-3p plays an oncogenic role. These findings indicate that miR-639, miR-641, miR-1915-3p, and miR-3613-3p can be used as new candidate biomarkers for CRC diagnosis. However, a functional evaluation of the dysregulation of these miRNAs remains to be elucidated in future studies.

## Figures and Tables

**Table 1:** Demographic, clinical and pathological features of patients with CRC

Characteristics	Patients (%)
**Age (years)**	
≥55	39 (66.1)
˂55	20 (33.9)
**Gender**	
Male	35 (59.3)
Female	24 (40.7)
**Cigarette smoking**	
Yes	17 (28.8)
No	42 (71.2)
**Alcohol drinking**	
Yes	5 (8.5)
No	54 (91.5)
**Tumor location**	
Colon	37 (62.7)
Rectum	22 (37.3)
**Invasion**	
T1+T2	13 (22)
T3+T4	46 (78)
**Neural invasion**	
Yes	11 (18.6)
No	48 (81.4)
**Lymphovascular invasion**	
Yes	16 (27.1)
No	43 (72.9)
**Distant metastasis**	
M0	49 (83.1)
M1	10 (16.9)
**Lymph node metastasis**	
N0	35 (59.3)
N1+N2	24 (40.7)
**Tumor stage**	
I-II	30 (50.8)
III-IV	29 (49.2)
**Tumor size (cm)**	
≤6	33 (55.9)
>6	26 (44.1)
**Tumor histological type**	
Adenocarcinoma	43 (72.9)
Mucinous adenocarcinoma	16 (27.1)

T1: Tumor invades submucosa, T2: Tumor invades muscularis propria, T3: Tumor invades through the muscularis propria into pericolorectal tissues; M0: No distant metastasis, M1: Metastases in more than one organ/site or the peritoneum; N0: No regional lymph node metastasis, N1: 1-3 pericolic lymph involvement N2:2-4 pericolic lymph involvement or perirectal involvement.

**Table 2:** The association between miRNA expressions and clinicopathological characteristics of CRC patients

Variables	miR-639 Expression			miR-1915-3p Expression			miR-3613-3p Expression		
	Low	High	*p* value	Low	High	*p* value	Low	High	*p* value
**Age (years)**									
≥55	19	20	0.07	13	26	0.380	34	5	0.653
˂55	17	3		9	11		19	1	
**Gender**									
Male	12	12	0.151	11	13	0.261	21	3	0.679
Female	24	11		11	24		32	3	
**Cigarette smoking**									
Yes	12	5	0.338	5	12	0.426	15	2	1.000
No	24	18		17	25		38	4	
**Alcohol drinking**									
Yes	4	1	0.639	1	4	0.641	4	1	0.427
No	32	22		21	33		49	5	
**Tumor location**									
Colon	19	18	0.049*	12	25	0.317	32	5	0.396
Rectum	17	5		10	12		21	1	
**Invasion**									
T1+T2	9	4	0.492	5	8	1.00	13	0	0.322
T3+T4	27	19		17	29		40	6	
**Neural invasion**									
Yes	5	6	0.310	5	6	0.731	9	2	0.310
No	31	17		17	31		44	4	
**Lymphovascular invasion**									
Yes	8	8	0.290	4	12	0.234	14	2	0.658
No	28	15		18	25		39	4	
**Distant metastasis**									
M0	32	17	0.166	19	30	0.729	44	5	1.000
M1	4	6		3	7		9	1	
**Lymph node metastasis**									
N0	23	12	0.372	14	21	0.603	33	2	0.212
N1+N2	13	11		8	16		20	4	
**Tumor stage**									
I-II	12	18	0.001*	12	18	0.661	28	2	0.424
III-IV	24	5		10	19		25	4	
**Tumor size (cm)**									
≤6	19	14	0.541	13	20	0.706	30	3	1.000
>6	17	9		9	17		23	3	
**Tumor histological type**									
Adenocarcinoma	25	18	0.458	14	29	0.218	37	6	0.176
Mucinous adenocarcinoma	11	5		8	8		16	0	

*p <0.05 was considered significant.

## Abbreviations

miRNA: microRNA
CRC: colorectal cancer
qRT-PCR: quantitative real time PCR
KEGG: kyoto encyclopedia of genes and genomes
PPI: protein-protein interactions
FunRich: functional enrichment analysis
3'UTR: 3' untranslated region
Ct: threshold cycle
STRING: search tool for the retrieval of interacting genes
